# Systematic Exploration of Molecular Mechanisms and Natural Herbal Therapeutic Strategies for Cancer Cachexia

**DOI:** 10.3390/cancers18010104

**Published:** 2025-12-29

**Authors:** Pengyu Han, Xingyu Zhou, Guomin Dong, Litian Ma, Xiao Han, Donghu Liu, Jin Zheng, Jin Zhang

**Affiliations:** 1 Department of Traditional Chinese Medicine, Tangdu Hospital, Fourth Military Medical University, Xi’an 710023, China; 2 Shaanxi Provincial Key Laboratory of Integrated Traditional Chinese and Western Medicine in Oncology Diagnosis and Treatment, Fourth Military Medical University, Xi’an 710023, China; 3 College of Traditional Chinese Medicine, Shaanxi University of Chinese Medicine, Middle Section of Century Avenue, Xianyang 712046, China; 4 Department of Thoracic Surgery, Tangdu Hospital, Fourth Military Medical University, Xi’an 710023, China

**Keywords:** cancer cachexia, molecular mechanisms, herb, systemic inflammation, muscle atrophy

## Abstract

Cancer cachexia (CC) is a multifactorial, multi-organ syndrome characterized by systemic inflammation, metabolic dysregulation, anorexia, and progressive depletion of skeletal muscle and adipose tissue. Despite its high prevalence among patients with advanced malignancies, effective therapeutic options remain limited. Recent studies have elucidated the molecular underpinnings of CC and the therapeutic potential of natural herbs, highlighting the involvement of central nervous system regulation, adipose tissue, immune responses, gut microbiota, skeletal muscle, and disruptions in anabolic–catabolic signaling pathways such as mTOR, UPS, NF-κB, and STAT3. Persistent inflammation induces E3 ubiquitin ligases (Atrogin-1/MuRF-1) through cytokines including IL-6 and TNF-α, thereby impairing muscle homeostasis, while suppression of anabolic cascades such as IGF-1/mTOR further aggravates muscle atrophy. The limited efficacy and adverse effects of synthetic agents like megestrol acetate underscore the value of herbal therapies as safer adjunctive strategies. Botanicals such as *Coicis Semen*, *Scutellaria baicalensis*, and Astragalus demonstrate anti-inflammatory and muscle-preserving activities by modulating NF-κB, IL-6, and oxidative stress signaling. Numerous investigations indicate that these herbs downregulate MuRF-1 and Atrogin-1 expression, enhance appetite, and attenuate muscle loss, though they exhibit minimal influence on tumor suppression. While promising, current evidence remains largely preclinical and mechanistic validation is incomplete. This review consolidates contemporary insights into CC pathogenesis and the bioactivity of herbal interventions, highlighting the need for translational research to bridge preclinical findings with clinical applicability.

## Simple Summary

Cachexia often occurs in many cancer patients at the terminal stage, which seriously affects their subsequent treatment and prognosis. Although currently commonly used clinical drugs have provided significant assistance, their limitations are also quite obvious. Many natural herbs possess the property of “medicine–food homology” and have a good safety profile, so incorporating them into the treatment of cachexia is highly valuable. Meanwhile, the multi-target characteristic of herbs enables them to target the immune–metabolic axis and alleviate cachexia.

## 1. Introduction

Human body movement and a wide range of physiological functions rely on the regulation of skeletal muscle. As the most abundant tissue in the body, comprising approximately 40% of total body weight, skeletal muscle is organized into bundles of muscle fibers formed through myogenesis [1,2,3]. Cachexia, a syndrome characterized by skeletal muscle loss, is commonly observed in the advanced stages of chronic diseases such as cancer, AIDS, COPD, heart failure, and chronic kidney disease [4]. Generally, cachexia associated with cancer is referred to as cancer cachexia (CC). These conditions often induce a range of physiological changes, including systemic inflammation, anorexia, anemia, and reduced hormone synthesis, in addition to muscle loss. CC cannot be fully reversed by conventional nutritional support [2,5,6,7]. The prevalence of CC is particularly high in cancer, especially in gastrointestinal cancers, with studies indicating that approximately 70% of cancer patients develop CC, also known as CC, and 15–40% of them die as a result [8]. Characterized by energy imbalance and excessive protein degradation, CC impairs skeletal muscle growth. Skeletal muscle mass homeostasis is regulated by two primary pathways: anabolism and catabolism [9,10]. Anabolism, associated with protein synthesis, contrasts with catabolism, which involves protein degradation. Important anabolic pathways include the mammalian target of rapamycin (mTOR) pathway, insulin, and insulin-like growth factor (IGF-1), while catabolic pathways include the ubiquitin-proteasome system (UPS), autophagic-lysosomal pathway (ALP), and lysosomal degradation [2]. An imbalance between these pathways leads to muscle loss and disruption of mass homeostasis. These disruptions are primarily caused by reduced nutrient intake and metabolic abnormalities in cancer patients [8,11,12].

A 10% body weight loss due to cancer is generally defined as CC, which can be classified into three stages based on physical indicators and overall condition: pre-CC, CC, and refractory CC. CC not only diminishes patients’ quality of life but also negatively impacts the efficacy of primary disease treatment, leading to adverse outcomes [13,14,15]. Unfortunately, the precise mechanism of CC remains incompletely understood, though inflammatory mediators are known to play a central role. For instance, elevated levels of interleukin 6 (IL-6), tumor necrosis factor-α (TNF-α), and C-reactive protein (CRP) have been observed in CC patients [16]. These pro-inflammatory cytokines not only directly activate the UPS but also amplify inflammation through the Janus kinase (JAK)-STAT3-NF-κB signaling axis, suggesting that anti-inflammatory treatment could mitigate the malignant progression of CC. Nonsteroidal anti-inflammatory drugs (NSAIDs), such as celecoxib, are commonly preferred [17]. These NSAIDs inhibit the production of pro-inflammatory cytokines like IL-6 and TNF-α. However, targeting inflammation alone addresses only a small part of the problem; due to the complex pathophysiology of CC, this approach is insufficient.

This, however, represents only a fraction of the broader issue and is insufficient to address the complex pathophysiological mechanisms of CC. The absence of standardized treatment protocols remains a significant challenge in managing CC, and many drugs believed to alleviate the condition have failed in phase III clinical trials [18]. A common flaw among these drugs is their focus on simple anthropometric or dietary metrics—such as body weight and food intake—while neglecting the underlying drivers of CC. ASCO’s review of therapeutic agents for CC highlights that a multimodal approach, involving nutritional interventions and moderate physical activity, is currently the most effective strategy [15,19,20]. Among the available treatments, megestrol acetate, an appetite stimulant frequently prescribed for CC, warrants particular attention. Although it effectively stimulates hunger, prospective studies show it does not preserve lean mass or improve quality of life. The drug functions by upregulating hypothalamic neuropeptide-Y signaling, resulting in a transient increase in caloric intake. However, this central mechanism fails to address cytokine-induced anorexia, prevent UPS activation, or activate anabolic protein synthesis pathways, leaving the cachectic process unabated. Furthermore, megestrol acetate carries significant toxicity, including an increased risk of thromboembolism and fluid retention, which further deteriorates the patient’s physical condition and daily functioning [21,22]. These recurring failures underscore a crucial point: monotarget drugs that focus solely on body weight, while ignoring the immune–metabolic cycle driving CC, are fundamentally ineffective. In contrast, multi-component botanicals have consistently demonstrated clinical benefits in complex diseases. Their favorable safety profile and low cost make herbal medicines a promising, though underutilized, option for CC [23,24,25,26]. For instance, plant-derived flavonoids reduce NF-κB signaling and lower pro-inflammatory cytokine production, while specific saponins activate mTOR, promoting protein synthesis and inhibiting proteolysis [27]. Unlike single-target drugs such as celecoxib or megestrol acetate, botanicals modulate both inflammatory and anabolic/catabolic pathways simultaneously, offering an integrated approach that more effectively addresses the metabolic imbalance underlying CC.

Notably, a single-cytokine approach is no longer viable. Non-small cell lung cancer (NSCLC) is primarily driven by IL-6, while pancreatic tumors predominantly release IL-8 and IL-1 [28]. Future therapeutic strategies must therefore align tumor type with its dominant inflammatory mediators via a cancer-specific cytokine map. In addition, inflammation represents only one facet of the CC pathology. Dissecting CC without addressing the neuro-endocrine axis, mitochondrial function, and gut–host interactions provides a partial, and thus misleading, understanding. The true driver is a self-reinforcing immune–metabolic loop that integrates these systems. Current therapies, such as celecoxib and megestrol acetate, continue to target rudimentary endpoints; the path forward involves combining the precision of synthetic targeted therapies with the polypharmacology of botanicals. To achieve this, pharmacokinetic monitoring of herbal preparations—an underexplored frontier—must be established, allowing for quantification of bioactive exposure and its link to therapeutic efficacy. By coupling high-throughput omics with clinical phenotyping, CC-specific biomarkers and risk-prediction models can first be identified, followed by data-driven discovery of herbal constituents that target newly mapped nodes in the immune–metabolic circuitry. This rational integration of molecular selectivity with botanical complexity holds the promise for the next generation of evidence-based, personalized anti-CC therapies.

It should be pointed out that the underlying molecular mechanisms of CC have been comprehensively elucidated in numerous existing review articles [29,30,31]. This review establishes the correlation between the gut microbiota-immune-metabolism-muscle axis and cancer cachexia, a topic that has been rarely addressed in previous reviews. Furthermore, it systematically summarizes the therapeutic effects of various natural herbal in cancer cachexia, thereby laying a more precise theoretical foundation for subsequent clinical translational applications.

## 2. The Development of CC Involves Multiple Organs

CC is not a disorder limited to a single tissue; rather, it represents a widespread, multi-organ metabolic failure that involves the central nervous system (CNS), immune system, adipose tissue, gut, and skeletal muscle. Reciprocal signaling across these organ-to-organ axes creates self-sustaining feedback loops that continuously promote catabolism and inhibit anabolism, leading to inevitable weight loss.

### 2.1. Central Nervous System

CC is a form of malnutrition secondary to cancer, characterized by weight loss, muscle wasting, anorexia, abnormal fat metabolism, chronic systemic inflammation, and dysbiosis in the gut microbiota [32]. The primary cause of malnutrition in CC is reduced food intake due to anorexia, with the CNS playing a central regulatory role in the body’s feeding behavior [33,34]. In addition to anorexia, insulin resistance and reduced levels of anabolic hormones, which are also regulated by the CNS, are significant contributors to malnutrition [35,36]. Recent studies have increasingly focused on the influence of the hypothalamic-pituitary-adrenal (HPA) axis on CC [37], highlighting the neuro-endocrine-immune triad as a central hub, where reciprocal crosstalk actively drives the cachectic process [38,39,40,41]. The hypothalamus plays a key role in regulating energy balance, with food intake relying on its transmission of biological signals [40,42]. Neurons that produce neuropeptide Y (NPY) and Agouti-Related Protein (AgRP) stimulate appetite, while Pro-opiomelanocortin (POMC) and Cocaine- and Amphetamine-Regulated Transcript (CART) neurons suppress it. An imbalance between the stimulation of NPY/AgRP neurons and POMC/CART neurons can result in anorexia nervosa [33,42,43]. In addition, structures like the nucleus of the solitary tract, pre-AgRP, dopamine, and hypothalamic serotonin have been shown to modulate food intake [44,45]. Inflammation plays a pivotal role in activating these neuronal pathways. Chronic inflammation elevates pro-inflammatory factors in the nervous system, leading to reduced stimulation of appetite-promoting NPY/AgRP neurons and increased activation of appetite-suppressing POMC/CART neurons [46]. Furthermore, central inflammation hyper-activates the HPA axis, stimulating the adrenal cortex to release norepinephrine. In a chronically inflamed environment, elevated levels of norepinephrine bind to β2-adrenergic receptors (β2-ARs) on macrophages, triggering a cAMP-PKA-CREB cascade that amplifies the transcription of IL-1β, TNF-α, and IL-6—thus perpetuating systemic inflammation [47,48]. Beyond cytokines, prostaglandin E2 (PGE2) and glucocorticoids from the HPA axis synergize to suppress appetite, tightening the grip of the cachectic cycle [46]. Gastric emptying and appetite are interrelated processes, with faster gastric emptying stimulating appetite [49]. In cancer patients, high levels of inflammatory factors are released due to tumor invasion, metastasis, and the effects of radiotherapy and chemotherapy [50]. These inflammatory factors can delay gastric emptying and suppress appetite [51]. Beyond inflammatory factors, parathyroid hormone-related protein (PTHrP) activates hypothalamic urocortins 2/3, influencing gastric emptying via the vagus nerve, thereby reducing food intake [52]. This mechanism has been validated in animal models, where modulation of PTHrP secretion reduces energy loss in mice with CC [8,53]. Cancer patients often experience insomnia, depression, anxiety, and other forms of mental stress, which can also negatively impact food intake [54,55]. Ghrelin, a 28-amino acid growth hormone released by the stomach during hunger, plays a key role in stimulating food intake and regulating energy balance by transmitting peripheral nutritional signals to the brain [56]. Once reaching the hypothalamus, ghrelin activates AMP-activated protein kinase (AMPK) via the Ca^2+^-CaMKKβ pathway in AgRP/NPY neurons. Additionally, ghrelin mitigates CC-driven lipolysis and muscle wasting and suppresses the secretion of pro-inflammatory cytokines, such as IL-10, IL-6, and TNF-α [57,58,59]. The NF-κB-mediated UPS signaling pathway plays a critical role in muscle loss [60,61,62], and ghrelin can inhibit this pathway, thereby reducing muscle loss [63]. Ghrelin also suppresses the expression of muscle wasting proteins, such as Muscle Atrophy F-box protein (Atrogin-1) and Muscle RING-finger 1 (MuRF-1), through the PI3K-Akt-mTOR signaling pathway [58]. Despite ongoing discoveries regarding the role of ghrelin in CC, its precise role in cachectic patients remains controversial, and further research is required to clarify the underlying mechanisms.

### 2.2. Fat

Fat serves as a crucial energy reserve in the human body and plays a significant role in maintaining energy homeostasis. The development of CC has been linked to abnormalities in adipose tissue (AT), and the depletion of fat contributes to weight loss and weakness [64]. AT is classified into two types: white AT (WAT) and brown AT (BAT), both of which are essential for metabolic stability [65]. BAT cells are smaller and contain numerous mitochondria, which house uncoupling protein 1 (UCP-1). This protein enables energy from mitochondrial oxidative phosphorylation to be released as heat instead of being used for ATP synthesis, thus increasing energy expenditure by accelerating fat breakdown. WAT can undergo a process known as “browning,” acquiring BAT-like characteristics and promoting a whole-body catabolic state. This phenomenon of WAT browning has been validated in both animal and human studies [66], activating BAT and UCP1 to promote calorie release. It has been reported that exocytosis of Lewis cells releases PTHrP, which interacts with the parathyroid hormone receptor (PTHR) and activates protein kinase A to induce UCP1 expression, thus enhancing WAT browning [67]. Inflammatory signals are also key drivers of WAT browning. In advanced stages of CC, fat pads are infiltrated by macrophages, eosinophils, and other immune cells. Single-cell profiling has shown that these leukocytes interact with adipocytes, driving the transcription of STAT3/UCP1 in both tissues [68]. Notably, STAT3 activation in fat triggers thermogenic heat loss, while the same transcription factor in muscle accelerates proteolysis—opposing effects that highlight the need for a tissue-selective STAT3 modulator to restore energy balance. Browning-induced free fatty acids (FFAs) cross the blood-brain barrier, stimulating hypothalamic urocortins 2/3, which delay gastric emptying and suppress appetite. This FFA-hypothalamic axis has been confirmed in cachectic models, though the specific FFA isomers responsible remain undefined. Additionally, FFAs activate the paraventricular nucleus (PVN)–sympathetic circuit, enhancing norepinephrine release, which then feeds back through the cAMP/PKA/CREB cascade to amplify systemic inflammation. A dual-organoid platform, integrating adipose and skeletal muscle slices, could now be employed to unravel how the immune–lipid–CNS–muscle network is reconfigured in CC and to screen therapeutics that uncouple thermogenesis from proteolysis [48,69].

### 2.3. Immune System

**Figure 1:**
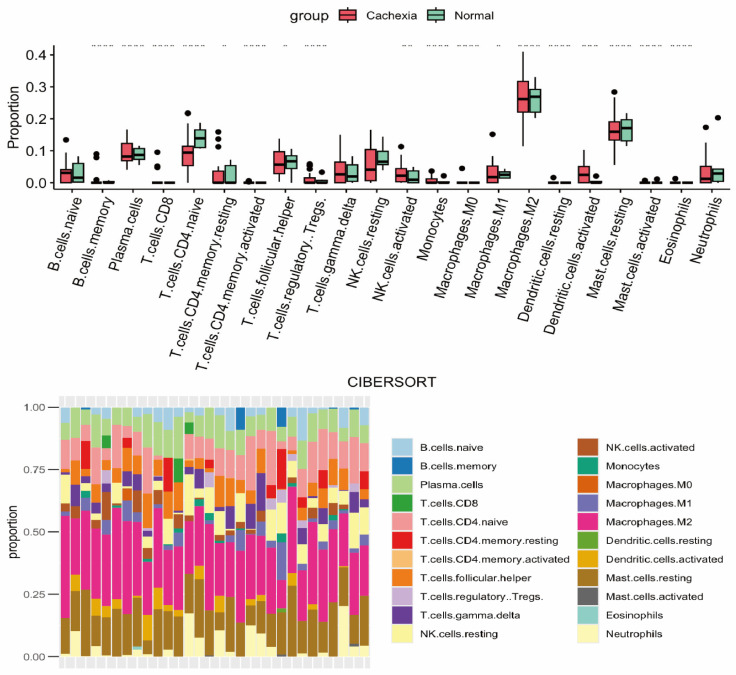
Immune infiltration in patients with cachexia. Boxplot of immune cells in patients with cachexia and immune cell accumulation in patients with cachexia.

The immune system plays a pivotal role in driving CC, with this process closely linked to the release of inflammatory factors. The UPS can be activated by TNF-α released from immune cells, leading to muscle atrophy. However, TNF-α is just one part of the broader picture: elevated levels of IL-6, IL-1, IL-8, and IL-10 in cancer patients converge on STAT3, enhancing energy expenditure and proteolysis, which exacerbates muscle wasting and intensifies the cachectic syndrome [70]. It is well established that E3 ligases in the UPS tag substrate proteins with ubiquitin (Ub) for degradation. Among these, Atrogin-1/MAFbx and MuRF-1 are muscle-specific E3 ligases whose transcription is driven by inflammatory cytokines such as TNF-α, IL-6, and IL-1. These cytokines activate relevant signaling cascades (e.g., NF-κB, STAT3), up-regulate MAFbx and MuRF-1, and accelerate the breakdown of contractile proteins, thus promoting muscle wasting [71]. The branched-chain amino acids (BCAAs) released during muscle breakdown overstimulate mTORC1, generating excess reactive oxygen species (ROS) and amplifying inflammation. In addition to providing pro-inflammatory substrates, elevated BCAAs epigenetically reprogram tumor-associated macrophages in coordination with cancer-derived CCL2 and CCL5, enhancing recruitment and shifting these cells toward a pro-cachectic phenotype [72]. Consequently, M2-like macrophages remain in a catabolic state, and their extensive infiltration sustains STAT3 activation, further accelerating muscle proteolysis [73,74]. M2 macrophages also release IL-6, TNF-α, and other inflammatory mediators that activate NF-κB, driving coordinated muscle and fat degradation through both the UPS and ALP, thereby perpetuating an immune-metabolic-muscle positive feedback loop. In addition to macrophages, T cells and myeloid-derived suppressor cells (MDSCs) have been identified as key players in CC. MDSCs increase whole-body oxygen consumption, a hallmark of CC, and fat pads from MDSC-high mice lose significantly more mass than those from MDSC-low littermates [75,76]. In contrast, T cells provide a protective effect: CD4+ Treg cells help preserve myofiber cross-sectional area and reduce atrophy. Given the complexity of the immune infiltrate, which spans multiple cell lineages, a new analysis of the public CC transcriptome dataset GSE34111 was conducted to map the complete immune landscape of CC, complementing existing literature [77]. This analysis revealed a higher expression of activated CD4+ cells and resting mast cells in cachectic patients (Figure 1).

Macrophage heterogeneity plays a pivotal role in driving CC through spatially distinct programs. The M2a subset, which is specific to CC, accumulates in atrophying muscle, where IL-6/TGF-β1 signaling activates STAT3–NF-κB pathways, and exosomal circTmeff1 impairs mitochondrial function, leading to myofiber wasting. M1 macrophages, which dominate the necrotic cores of tumors, release TNF-α to activate the UPS and recruit neutrophils that further amplify protein degradation. M2c macrophages patrol the invasive margin, suppress T-cell activity, and facilitate tumor progression, indirectly exacerbating CC. In parallel with these macrophage subsets, Treg cells—significantly expanded in both peripheral blood and the tumor microenvironment of cachectic patients—form immunosuppressive micro-islands around M2a macrophages. This interaction sustains chronic inflammation and results in IL-35 secretion, which impairs insulin signaling and induces autophagy in myocytes, further accelerating muscle loss.

However, much of this immune circuitry has been defined in rodent models, with limited validation in humans, and cancer-type specificity remains poorly understood. This highlights the urgent need for multi-omic integration to fully decode the immune-regulatory network driving CC [78,79].

### 2.4. Gut and Microbiota

The inner lining of the gastrointestinal tract serves as a barrier, separating luminal contents—which include a diverse community of bacteria, fungi, viruses, and other microbes—from the internal milieu. Current evidence indicates that the gut microbiota plays a critical role in modulating host nutrient absorption while simultaneously acting as a biological barrier, recognizing and repelling foreign microorganisms to maintain intestinal integrity [80,81,82]. Research has shown that the intestinal mucosa is inevitably compromised in cancer [83], with rapid tumor growth, radio-chemotherapy, and an influx of pro-inflammatory cytokines all contributing to the breakdown of epithelial integrity and increased permeability. This damage alters the gut microbiota, allowing bacterial endotoxins and lipopolysaccharide (LPS) to translocate into the bloodstream, where they bind to Toll-like receptor 4 (TLR4) and induce the massive production of inflammatory cytokines, thereby triggering systemic chronic inflammation and increasing the risk of CC [84]. The ApcMin/+ mouse model has solidified this connection. As tumors advanced, mice progressively lost weight, and a hydrophilic permeability tracer revealed gut leakiness that correlated with the severity of CC. Cytokine levels rose in parallel with barrier dysfunction, and at the terminal stage, endotoxin levels were five times higher, accompanied by insulin resistance and disrupted lipid metabolism. Human data mirror these findings. In gastric adenocarcinoma patients, claudin was upregulated while occludin was downregulated, microbial diversity collapsed, bacterial translocation increased, and systemic inflammation escalated. Additionally, levels of IL-6 and lipopolysaccharide-binding protein (LBP) surged. Neutralizing IL-6 in the CT26 colon cancer model restored microbial balance and eubiosis, while elevated serum LBP and IL-6 emerged as robust biomarkers that can stratify prognosis and guide diagnosis and therapy for CC [85,86]. In recent years, research attention has increasingly shifted to the liver-gut axis in the field of cachexia, and the dysregulation of this axis has been identified as a crucial contributor to the pathogenesis of cachexia. Whether it is hepatocellular carcinoma (HCC), other liver diseases, or other pathological conditions that induce hepatic injury, they all promote the overgrowth of pathogenic bacteria in the intestine [87]. Previously, several studies have reviewed the impacts of intestinal barrier dysfunction and gut microbiota dysbiosis on cytokine profiles in HCC. HCC can induce the massive production and release of LPS, a consequence primarily attributed to hepatic dysfunction, hepatic portal hypertension, and abnormal metabolism of total bile acids. These pathological alterations collectively impair the intestinal mucosa, thereby facilitating LPS translocation into the systemic circulation; subsequent activation of the LPS- TLR4- NF-κB signaling axis ultimately contributes to the development of cachexia. [88,89]. Malaise, often closely linked to inflammation, is further exacerbated by changes in gut flora, leading to altered bowel habits. Frequent diarrhea, for example, can result in electrolyte imbalances and nutrient loss, while the abundance of intestinal flora also shifts [82].

**Figure 2:**
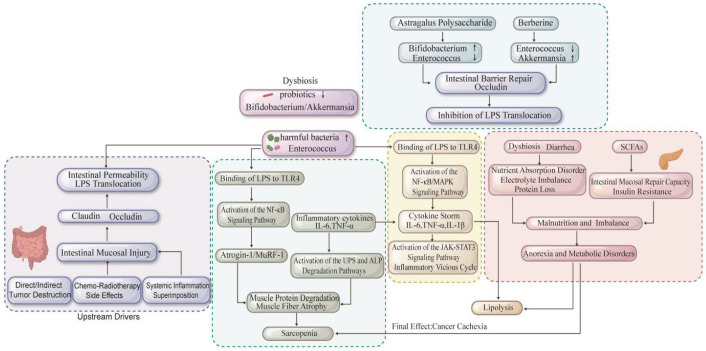
The mechanism of action and intervention targets of the “gut microbiota-immune-metabolic-muscle” axis in cancer cachexia. Specifically, factors like cancer induce intestinal mucosal injury, elevated intestinal permeability, and gut dysbiosis (characterized by a decrease in beneficial bacteria and overproliferation of harmful bacteria such as Enterococcus). Harmful bacteria or translocated lipopolysaccharide (LPS) activate Toll-like receptors (TLRs) and NF-κB pathway, which induces the release of inflammatory cytokines and activates muscle protein degradation pathways (UPS and ALP). This upregulates Atrogin-1 and MuRF-1 to trigger muscle atrophy. Meanwhile, intestinal dysfunction exacerbates malnutrition and metabolic abnormalities, thereby promoting the progression of cachexia. In contrast, beneficial bacteria including Bifidobacterium and *Akkermansia muciniphila*, as well as herbal components such as astragalus polysaccharide and berberine, can interrupt the vicious cycle of cachexia by modulating the gut microbiota, repairing the mucosal barrier, and inhibiting inflammatory and muscle degradation pathways.

Muscle wasting, the hallmark of CC, has directed focus onto the immune–gut–metabolic–muscle axis. Skeletal muscle acts as an endocrine organ, exchanging molecular signals with distant tissues. Early studies found that broad-spectrum antibiotics in mice led to a decline in grip strength, while re-colonization restored it. In C2C12 myotubes, LPS alone was sufficient to drive the secretion of TNF-α and IL-6, which in turn upregulate Atrogin-1 and MuRF-1, triggering muscle atrophy, thus linking a compromised gut directly to proteolysis. Taxonomic analysis reveals that different microbes have opposing effects. Bifidobacterium, a core beneficial genus, reduces systemic inflammation and improves functional outcomes. *Akkermansia muciniphila* strengthens the epithelial barrier by increasing occludin expression, curbing LPS translocation, while Enterococcus amplifies inflammation and circulating LPS, accelerating CC. Active compounds from medicinal herbs can help interrupt this vicious cycle. In Lewis-lung-cachectic mice, astragalus polysaccharide doubled the abundance of Bifidobacterium, reduced Enterococcus by 60%, suppressed NF-κB activity, and repressed the expression of Atrogin-1 and MuRF-1. In the CT26 colon-cancer model, berberine reduced Enterococcus, expanded Akkermansia, protected the mucosal barrier, and reduced LPS-driven inflammation. These data establish a clear gut–muscle connection in CC, and future metagenomic and single-cell spatial transcriptomic approaches will further decode how the microbiota governs this multi-organ axis [15,90,91] (Figure 2).

TNF-α, tumor necrosis factor α; IL-1, interleukin 1; IL-6, interleukin 6; IL-8, interleukin 8; IL-10, interleukin 10; UPS, protein-ubiquitinosome system; LPS, lipopolysaccharide; TLRs, Toll-like receptors.

### 2.5. Skeletal Muscle

Skeletal muscle is crucial for maintaining normal physiological functions, and its loss is a key feature of CC, significantly impacting patients’ quality of life. Studies indicate that the surface area of skeletal muscle in cachectic patients is reduced by 33% compared to healthy individuals, with the skeletal muscle index decreasing by approximately 13% [92]. Activation of the UPS, ALP, and Ca^2+^ pathways is the primary mechanism involved in muscle protein degradation [93,94,95]. The UPS pathway, the most critical for protein degradation, plays a central role in the induction of muscle atrophy. E3 ligases within the UPS system recognize specific target proteins for degradation, with this process modulated by multiple signaling pathways, including PI3K-AKT, NF-κB, and MAPK [95,96,97]. ALP is another protein degradation pathway, primarily responsible for eliminating misfolded proteins, regulating key mTOR and AMPK activators, and maintaining intracellular energy balance [93,98]. Tumor cells, along with the abundant M2 macrophages they recruit, consistently produce inflammatory cytokines [73]. These ligands activate gp130, triggering JAK2-STAT3 phosphorylation, which suppresses IGF1-PI3K-AKT signaling, derepresses FOXO3, and initiates autophagy, ultimately leading to widespread protein degradation and the onset of CC. In patients, this is reflected by increased autophagy, with significant upregulation of both BNIP3A and LC3B [98]. Autophagy also reshapes glucose metabolism by downregulating GLUT4, inhibiting key glycolytic enzymes, and forcing muscle cells to rely on fatty acid oxidation [99]. This shift generates excessive ROS and activates inflammasomes, which in turn amplify the inflammatory response. Ca^2+^ plays a pivotal role in regulating muscle function, and abnormalities in Ca^2+^ concentrations can directly impair muscle function [94]. Although Ca^2+^ involvement in muscle proteolysis related to CC has been infrequently reported, it has been shown to be involved in dexamethasone-induced muscle atrophy [100]. Ca^2+^ regulates the binding of calpastatin to calpain [100], and in a rat model, elevated levels of calpain were observed in aged rats, with inhibitory changes in calpastatin [101]. A malignant stroma model of hepatocellular carcinoma revealed high activation of the Ca^2+^-dependent protein hydrolysis pathway, a process linked to Ca^2+^ accumulation induced by the protein hydrolysis-inducing factor (PIF), which leads to skeletal muscle degradation [102].

**Figure 3:**
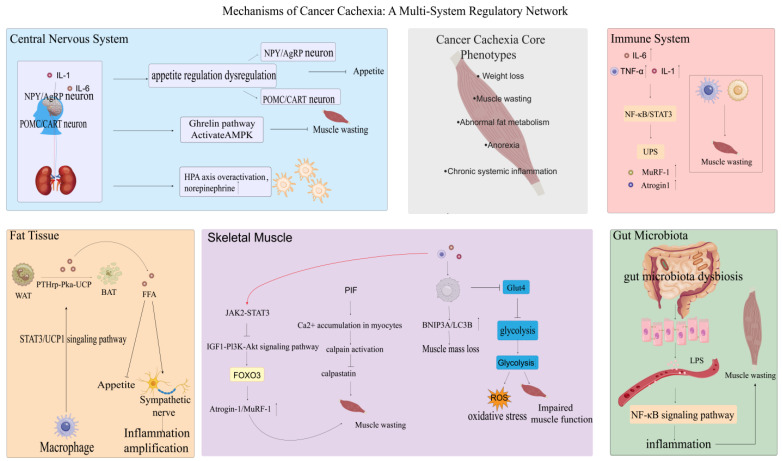
CC involves multiple organs. The image illustrates the effects on various organs during CC and the underlying mechanisms. Inflammatory factors released by cancer directly impact muscles, while in the nervous system, these factors influence the CNS, disrupting the hypothalamo-pituitary-adrenal axis. This results in insufficient secretion of gastric starvation hormones and delays gastric emptying, impairing food intake. Inflammatory factors also cause the browning and breakdown of WAT and alter immune cell function, contributing to muscle and fat loss. Cancer-induced damage to the intestinal mucosa allows endotoxins to enter the bloodstream, further promoting muscle and fat loss. Endotoxins from cancer in the circulation trigger systemic inflammation.

To intuitively demonstrate the characteristics of cancer cachexia (CC) as a trans-organ syndrome, we have constructed a mechanism diagram involving major organs (Figure 3).

## 3. Metabolic Dysregulation in CC

### 3.1. Anabolism

The primary component of skeletal muscle is protein, and enhancing protein synthesis while reducing protein degradation is critical for alleviating muscle atrophy. Skeletal muscle homeostasis is regulated by a balance between anabolic and catabolic pathways, and cancer disrupts this balance. Anabolism, which promotes muscle growth by synthesizing proteins, primarily relies on pathways such as mTOR and insulin. mTOR, a key nutrient regulator [103], consists of two complexes: mTORC1, which governs anabolism, and mTORC2, which primarily regulates glucose and lipid homeostasis [104]. mTORC1 stimulates anabolism via the 4E-binding protein (4E-BP) signaling pathway. Specifically, mTORC1 activates the phosphorylation of S6 kinase and the eukaryotic translation initiation factor 4E-BP, promoting protein synthesis. Dysregulation of mTORC1 expression during muscle growth can result in muscle atrophy, as mTORC1 also influences muscle ALP [105,106]. One study found that the absence of mTORC1 impaired muscle development and slowed growth in young mice. However, in adult animals, mTORC1 is not the sole pathway for protein synthesis; a significant portion of protein synthesis occurs independently of mTORC1. In adult humans, mTORC1 appears to play a minimal role in maintaining muscle homeostasis [107]. This discrepancy likely arises from the limited human tissue data available and the extrapolation of juvenile findings to adults, which may distort the therapeutic potential of mTOR modulation [108]. This suggests that mTORC1 is crucial for muscle growth in young animals but plays a more regulatory, fine-tuning role in adults. This insight offers a new perspective on understanding both physiological muscle development and pathological muscle wasting in CC. Inflammation is a primary factor in mTOR suppression. Elevated IL-6 levels in CC activate AMPK, which in turn inhibits mTOR expression. Conversely, over-activation of mTOR leads to oxidative damage, as demonstrated in a model of aged mice. The mechanisms behind mTOR over-activation remain unclear, though inflammation and protein denaturation are potential contributing factors [109].

IGF1 and insulin are also pivotal in the development of CC [110]. In normal muscle tissues, the IGF1/mTOR signaling pathway promotes protein synthesis; however, in CC, this pathway is inhibited, reducing the phosphorylation of key proteins like AKT and GSK3, and ultimately impairing protein synthesis. IGF1 can promote myotube differentiation and prevent muscle atrophy. In cachectic patients, however, circulating IGF1 levels are low. Administration of IGF1 has been found to activate the IGF1-PI3K-AKT pathway, reducing the expression of MuRF-1 and Atrogin-1, which in turn mitigates muscle atrophy [111]. Insulin plays a role in regulating muscle protein hydrolysis, and its administration in septic rats reduces UPS activation, thereby decreasing muscle protein degradation [112]. In diabetic patients, insulin resistance leads to the over-activation of the UPS system, contributing to skeletal muscle atrophy [113]. Insulin resistance also occurs in patients with CC [112], possibly due to TNF-α, which blocks the phosphorylation of the insulin receptor substrate (IRS), impairing insulin signaling [114]. In this insulin-resistant state, the phosphorylation of the PI3K-AKT pathway is reduced, leading to decreased release of FOXO and caspase-3, while increasing protein hydrolysis activity. This highlights the importance of maintaining insulin sensitivity in the resolution of CC [115].

The mTOR anabolic blockade does not function in isolation. IL-6, TNF-α, and other cytokines activate AMPK, which then directly inhibits mTORC1. This cytokine-AMPK–mTOR axis represents a metabolic fulcrum, where immune signals convert prolonged anabolic suppression into a catabolic state. In CC, mTORC1 activity is notably low, indicating a bidirectional imbalance in which both protein synthesis and degradation pathways are dysregulated, rather than a defect in a single pathway. Astragaloside IV, a purified extract of *Astragalus membranaceus*, alleviates this suppression by reactivating PI3K-AKT, restoring mTORC1 signaling, and reducing IL-6-driven AMPK phosphorylation, thus accelerating protein synthesis. In the same study, a combination of astragaloside IV with a small-molecule mTOR agonist promoted C2C12 myotube formation more effectively than either agent alone. Given the limited target specificity of synthetic drugs, a herbal-pharmacological co-therapy platform offers a promising avenue for clinical translation [116,117].

### 3.2. Catabolism

Anabolism is responsible for protein synthesis, while catabolism drives protein degradation. As previously discussed, the malignant phase of CC is marked by an initial decrease in protein synthesis followed by an increase in protein catabolism, with inflammation being the primary driver of these changes [15,32]. In CC, muscle growth inhibitors from the TGF-β family, which act as negative regulators of muscle growth, play a significant role in muscle loss by inhibiting the Akt-mTOR signaling pathway, thus suppressing protein synthesis [118]. Mice deficient in muscle growth inhibitors exhibit increased muscle mass [119]. Activin A, which is elevated in cancer patients, along with muscle growth inhibitors, upregulates the expression of MuRF-1 and Atrogin-1, leading to muscle degradation [120]. Furthermore, muscle growth inhibitors and activin A bind to ActR2B, triggering the activation of two transcription factors, SMAD2 and SMAD3, which result in the upregulation of Atrogin-1 [121]. Inflammation also impacts myoinhibin expression, which is elevated during chronic inflammation. Targeting muscle growth inhibitors has emerged as a promising strategy for CC drug development, though the exact mechanisms require further clarification [122].

Activation of the NF-κB, TNF-α, and IL-6-JAK-STAT3 signaling pathways is crucial for the development of CC [123,124,125], all of which are linked to muscle atrophy. NF-κB responds to TNF-α signals, and in CC, it inhibits the expression of MyoD, a key regulator of muscle formation, thereby hindering muscle development and promoting muscle degradation [126]. In addition to TNF-α, NF-κB can induce protein hydrolysis through other pathways, such as the iNOS-NO pathway, which is also implicated in muscle atrophy [126]. Beyond modulating NF-κB, TNF-α, an inflammatory cytokine released by macrophages, drives skeletal muscle breakdown via the UPS pathway, leading to muscle degradation. TNF-α stimulates the high expression of Atrogin-1 in myoblasts, activates the p38-AMPK pathway, and induces muscle catabolism [127]. STAT3 plays a critical role in various muscle-associated cells and regulates myofibrils to control skeletal muscle mass. Inflammatory factors are key to STAT3 activation, with the IL-6-JAK signaling pathway promoting STAT3 phosphorylation, which increases MAFbx and MuRF-1 expression. This leads to protein hydrolysis and muscle atrophy, and STAT3 further activates the NF-κB signaling pathway to induce apoptosis, contributing to muscle loss [128]. While these mechanisms are becoming clearer, further validation through extensive experimentation is needed.

**Figure 4:**
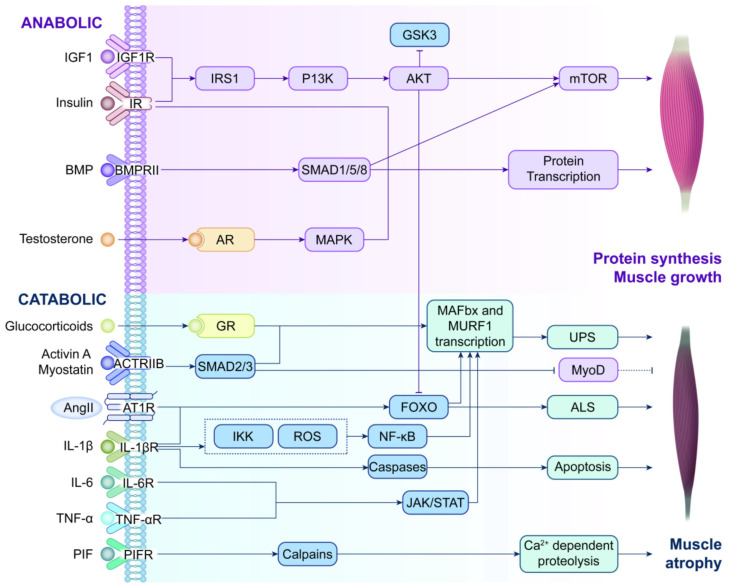
Muscle mass homeostasis is bidirectionally controlled by anabolic and catabolic cues. Nutrients and growth factors stimulate the PI3K-AKT-mTOR pathway, in cooperation with MAPK and BMP-SMAD1/5/8 axes, to enhance transcription and protein synthesis. In cachexia, tumor- and immune-derived inflammatory cytokines activate NF-κB, upregulating UPS and ALS pathways. Additionally, activin/myostatin-ActRIIB-SMAD2/3, glucocorticoid-GR, and AngII-AT1R pathways similarly activate UPS/ALS, collectively driving myofibrillar degradation. Dashed lines indicate inhibited routes.

The TNF-α–NF-κB–STAT3 axis is not merely a downstream executor of proteolysis. Activated STAT3 trans-activates IL-6 transcription, establishing a self-amplifying IL-6–pTyr705-STAT3–pSer727-STAT3–IL-6 loop that perpetuates inflammatory catabolism. In cachectic mouse models, muscle-specific deletion of STAT3 significantly reduces UPS activity and slows protein degradation, illustrating the close link between immune signaling and proteolytic metabolism. Unlike the mTORC1-mediated anabolic pathway, the NF-κB/STAT3 cascade is highly sensitive to nutrient scarcity: under protein-deficient conditions, UPS is activated within hours, while mTORC1 requires more than 72 h of sustained starvation to significantly reduce synthetic rates. This discrepancy provides a therapeutic window for initiating NF-κB/STAT3 blockade early in at-risk or pre-cachectic patients. Interestingly, NF-κB inhibition alone does not fully prevent weight loss in adult CC patients, though it is effective in murine models, indicating that multi-target, multi-pathway interventions will be necessary. Licochalcone A, a bioactive flavonoid from licorice, exemplifies this strategy by simultaneously blocking NF-κB-p65 nuclear translocation and STAT3 phosphorylation, offering a single-agent, dual-target approach for JAK–STAT3-driven pathologies [128,129] (Figure 4).

### 3.3. Emerging Regulators

Beyond the canonical NF-κB, STAT3, and AMPK pathways, the field of CC is evolving with the integration of two novel layers—non-coding RNAs and the mitochondria–inflammation axis—whose roles within the immune-metabolic framework are still largely unexplored. These emerging elements are now being incorporated into the broader catabolic/anabolic network.

Circular and exosomal non-coding RNAs act as long-range metabolic regulators. In WAT, circPTK2 is significantly upregulated, sponging miR-182-5p to relieve the repression of JAZF1, thereby accelerating lipolysis. In skeletal muscle, circDdb1 translates into an 867-amino acid peptide that binds to eukaryotic elongation factor 2, increases Thr56 phosphorylation, and globally stalls protein translation. Additionally, circANAPC7 sequesters miR-373, de-represses PHLPP2, and drives muscle wasting through the AKT-TGF-β pathway. Tumor-derived extracellular vesicles (EVs) add a remote-regulation dimension: EV-miR-223-5p, exported from pancreatic ductal adenocarcinoma, targets the 3′UTR of MAFA and down-regulates METTL14, creating an m6A-deficient epigenetic environment that enhances MuRF-1 transcription and proteolysis [130,131].

Mitochondria, once regarded solely as energy factories, are now recognized as signaling organelles. In CC, mitochondrial cristae become fragmented, ATP output collapses, and the resulting ROS/IL-6/TNF-α burst fuels NF-κB p65 nuclear translocation, which up-regulates MuRF-1 and Atrogin-1. Simultaneously, the leakage of damaged mtDNA triggers the STING–TBK1 cascade, which heightens STAT3 phosphorylation and inhibits mTORC1-dependent protein synthesis. Pharmacological blockade of STING in Lewis-lung-cachectic mice significantly attenuates muscle loss, highlighting the mitochondria–inflammation axis as a potential druggable checkpoint downstream of immune-metabolic stress [132].

## 4. Natural Herbs for Cancer Malignancy Treatment

**Figure 5:**
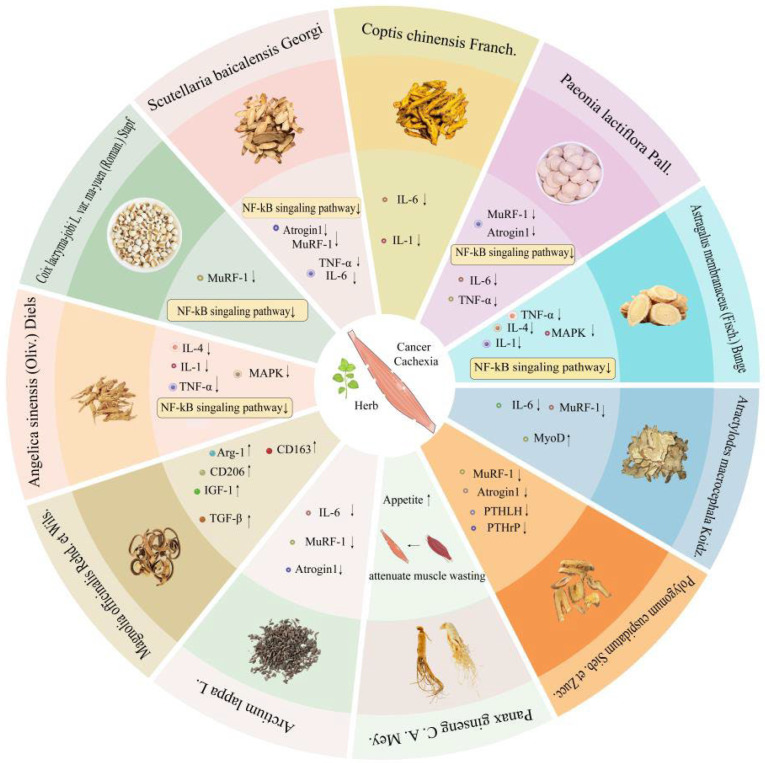
Circular Diagram of Pharmacological Characterization and Mechanism of Action of TCM Intervention Against Cancer Cachexia. It exhibits the physical pharmacological characterization of TCM, clarifies the material forms of the interventional agents, and meanwhile establishes systematic correlations between different TCM and the key molecular targets, signal transduction pathways as well as functional effects involved in the pathological processes of cancer cachexia.

As a multi-organ syndrome, the pathogenesis of CC remains incompletely understood, and current treatments are limited to single-agent therapies. [15]. The use of herbal medicine is widespread in East and Southeast Asia and has shown promise as a complementary treatment for various diseases. [133,134,135]. If further research confirms their effectiveness, these herbs may offer a novel approach for treating CC. (Table 1) (Figure 5).

### 4.1. Coix lacryma-jobi L. var. ma-yuen (Roman.) Stapf

*Coicis Semen*, the mature and dried seed of *Coix lacryma-jobi* L. *var. ma-yuen (Roman.) Stapf*, is a traditional medicinal herb commonly used as both food and medicine. In traditional Chinese medicine (TCM), *Coicis Semen* is commonly utilized for its efficacy in invigorating the spleen and promoting diuresis, as well as reducing swelling and expelling pus. The kernel serves as the core active part of this herb. Modern pharmacological studies have confirmed that the kernel contains a diversity of bioactive components, with substantial variations in component contents across different anatomical parts of the seed [146]. Specifically, the levels of coixenolide, coix protein and squalene in the kernel are significantly higher than those in the seed coat and endosperm, whereas polysaccharides and flavonoids are preferentially enriched in the inner-layer tissues of the kernel. These bioactive constituents collectively constitute the material basis underlying the pharmacological actions of *Coicis Semen*. Mechanistically, coixenolide exerts direct anti-tumor activity by inhibiting the signaling pathways implicated in tumor cell proliferation, while squalene mitigates oxidative stress-induced damage through enhancing the activity of antioxidant enzymes [146,147,148]. Research has demonstrated that *Coicis Semen* oil, derived from the seeds, exhibits significant efficacy in treating various cancers. Beyond cancer treatment, *Coicis Semen* oil has also been shown to alleviate cancer-related symptoms such as pain and CC. To evaluate its effectiveness in improving CC, researchers established a cancer cachexia model using Lewis lung cancer -bearing C57BL/6 mice, and administered *Coicis Semen* oil at a conventional dosage of 2.5 mL/kg/day for 24 consecutive days. The results indicated that *Coicis Semen* oil improved cancer-induced weight loss and increased muscle strength. The main components of *Coicis Semen* oil include coix seed lipids, coixin, and squalene, which are believed to possess anti-inflammatory and anti-aging properties. The mechanism through which *Coicis Semen* oil alleviates CC primarily involves reducing systemic inflammation by lowering serum levels of inflammatory cytokines such as IL-6 and TNF-α. Additionally, *Coicis Semen* oil reduces the phosphorylation of p65, a marker of NF-κB activation, inhibiting the NF-κB signaling pathway and decreasing the expression of MuRF-1. Moreover, *Coicis Semen* oil lowers AMPK phosphorylation levels, contributing to the reduction in muscle loss [136,149]. Tritium-tracer studies reveal that after administration of *Coicis Semen* oil micro-emulsion via intragastric or tail-vein injection in rats, the drug is eliminated slowly, with t½ of 15.84 h intravenously (i.v.) and 14.23 h orally (p.o.). The oral bioavailability is 62% of the i.v. value. Following i.v. administration, the emulsion is rapidly distributed, with the highest concentrations found in the liver and spleen. Within 24 h, approximately 38% of the dose is recovered in urine and 60% in feces, resulting in a total recovery of about 40%. Plasma-protein binding is 98% in vitro and 81% in vivo.

In clinical translational application, Kanglaite Injection (KLT), with coix seed oil as the primary active component, has been adopted for adjuvant therapy of clinical malignancies. A clinical trial revealed that when Stage IV lung cancer patients were administered Kanglaite Injection at a dosage of 200 mL per day for 21 consecutive days, their average body weight increased by 55.9% compared with the baseline before treatment. In clinical practice, occasional adverse reactions such as allergic responses and gastrointestinal discomfort have been reported. Currently, there is a paucity of safety data pertaining to the clinical use of *Coicis Semen* oil, which necessitates further in-depth investigation for clarification [150,151].

Despite showing clear benefits in rodent models of CC, large-scale clinical trials are still lacking. Currently, the extract is used as an adjunct in cancer therapy, rather than as a registered agent for CC. However, accumulating data suggest its therapeutic potential warrants further investigation.

### 4.2. Scutellaria baicalensis Georgi

*Scutellaria baicalensis Georgi* is the rhizome of the medicinal plant *Scutellaria baicalensis Georgi*, known for its therapeutic properties in treating various diseases. In TCM, *Scutellaria baicalensis Georgi* is credited with the efficacies of clearing heat and drying dampness, purging fire and detoxifying, as well as stopping bleeding and preventing miscarriage. Modern pharmacological research has verified that the medicinal value of *Scutellaria baicalensis Georgi* derives from the abundant bioactive components in its rhizomes, where the content of bioactive constituents is significantly higher than that in other plant parts such as stems and leaves; among these components, baicalin serves as the core bioactive ingredient. In addition to baicalin, the rhizomes also contain a variety of flavonoid bioactive components including baicalein, wogonin, wogonoside and oroxylin A. These components synergistically enhance its pharmacological activity: for instance, baicalein and baicalin can jointly inhibit the release of inflammatory cytokines, while wogonin potentiates the anti-tumor efficacy of baicalin [152,153,154]. As a flavonoid compound, baicalin has been the subject of extensive structure-activity relationship (SAR) studies, which have demonstrated that the substitution position of hydroxyl groups on the parent nucleus of baicalin is critical to its anti-inflammatory and anti-tumor activities. Specifically, the conjugation of the 7-position hydroxyl group with glucuronide enhances its water solubility and in vivo metabolic stability, whereas the presence of the 5-position hydroxyl group markedly improves its inhibitory activity against inflammatory signaling pathways [155]. In cancer, *Scutellaria baicalensis Georgi* has demonstrated the ability to inhibit tumor growth and angiogenesis. In recent years, its positive effects on CC treatment have been increasingly recognized, with baicalin identified as the key active compound for alleviating CC. In a study on malignant muscle atrophy in CT26-loaded mice, researchers administered baicalin via intragastric gavage at doses of 50 mg/kg and 150 mg/kg, once daily for 21 consecutive days. The results demonstrated that baicalin in the high-dose groups improved feeding behavior, reduced serum levels of inflammatory cytokines such as IL-6 and TNF-α, decreased the expression of Atrogin-1 and MuRF-1, and lowered the phosphorylation of p65. These findings suggest that baicalin reduces NF-κB signaling pathway activation by decreasing cancer-related inflammatory factors. Platinum-based chemotherapy agents, which are commonly used in cancer treatment, have also been linked to the development of CC, as prolonged chemotherapy increases inflammatory factor levels. In a mouse model of lung cancer, cisplatin treatment led to a 20% loss in body weight, but baicalin administration improved body weight without causing any additional adverse effects. A randomized controlled trial of oral baicalin in humans demonstrated rapid absorption, with a Tmax of approximately 3 h and t½ ranging from 7.8 to 14.9 h. Cmax ranged from 280 to 629 ng/mL, and the AUC increased with doses up to 800 mg, although unchanged drug excreted in urine was less than 1%. Metabolite exposure was similar to or exceeded that of the parent compound, with levels of 7BS and BGG 5 to 40 times higher. A third metabolite showed comparable exposure. All metabolites exhibited biphasic urinary excretion, with peaks at around 2 h and between 12 and 24 h. Cumulative 72-h recovery of metabolites was between 7% and 16% [137,156,157]. Due to the paucity of clinical research data on *Scutellaria baicalensis Georgi* or its active component baicalin, the toxicity and adverse effects of these agents in humans remain unclear. Nevertheless, they have exhibited favorable safety profiles in animal models.

### 4.3. Coptis chinensis Franch

Coptidis Rhizoma, the rhizome of *Coptis chinensis Franch.*, is widely used in East and Southeast Asia for its efficacy in ameliorating inflammation, treating diabetes, and managing digestive disorders. It contains various alkaloids, with berberine being the most pharmacologically active component. Modern pharmacological research has confirmed that the pharmacological activities of Coptidis Rhizoma. are attributed to the abundant alkaloid components in its rhizomes, where the content of bioactive constituents is significantly higher than that in other plant parts such as leaves and stems. Among these components, berberine serves as the core active ingredient with the most potent pharmacological activity. In addition to berberine, the rhizomes of Coptidis Rhizoma also contain a variety of isoquinoline alkaloids including coptisine, palmatine, jatrorrhizine and epiberberine [158]. These components exert a synergistic effect to enhance its overall pharmacological activity: for instance, coptisine and berberine can synergistically inhibit the release of inflammatory cytokines, while palmatine can assist berberine in regulating the balance of intestinal flora, thereby further potentiating the anti-inflammatory efficacy [159,160]. As an isoquinoline alkaloid, berberine has been extensively investigated in SAR studies. These studies have demonstrated that the 7,8-dimethoxy substitution on the parent nucleus of berberine is the key site mediating its anti-inflammatory activity, whereas the presence of the hydroxyl group at the 9-position can improve its water solubility. Furthermore, structural modification of berberine can markedly enhance its bioavailability as well as its anti-inflammatory and anti-tumor activities [161].

Coptidis Rhizoma can alleviate CC. To verify this efficacy, researchers administered Coptidis Rhizoma and berberine via dietary incorporation at respective doses of 10 mg and 20 mg for Coptidis Rhizoma, and 1 mg and 4 mg for berberine, followed by a consecutive observation period of 14 days. Results showed that this intervention reduced weight loss and preserved the normal morphology of muscle and AT in the CT26 tumor-bearing mouse model. These effects are primarily attributed to its ability to lower the levels of inflammatory cytokines such as IL-1 and IL-6 in the serum. This was similarly observed in a CC model of esophageal cancer, where Coptidis Rhizoma treatment reduced weight loss, though it did not effectively control tumor volume. Therefore, the therapeutic mechanism of Coptidis Rhizoma in treating CC appears to be its ability to modulate the systemic inflammatory response. Further development and in-depth studies are needed to explore the full potential of this treatment in the future. In a phase-I randomized trial of berberine for colon cancer, the alkaloid significantly reduced IL-6, TNF-α, and IL-8, confirming its clinically relevant anti-inflammatory activity. Pharmacokinetic analysis showed poor gastrointestinal absorption and negligible bioavailability: after a single 400-mg oral dose, the mean Cmax was approximately 0.4 ng/mL, and the AUC_0_–∞ was around 9.18 h·ng/mL. The drug distributed widely to the liver, kidneys, and other organs, with tissue levels exceeding plasma concentrations. However, almost all of the dose was recovered unchanged in feces, with less than 1% excreted as the parent compound in urine. Extensive first-pass metabolism further limits systemic exposure, explaining the low plasma concentrations despite high oral doses [138,162].

In cancer cachexia research, Coptidis Rhizoma and its extract berberine have demonstrated promising anti-inflammatory and muscle-protective potential. However, research investigating the risks associated with their long-term administration remains relatively scarce. This indicates that while clinical translation can be pursued to a certain extent, large-scale, rigorous, and scientifically sound clinical trials are still required to establish their safety profile.

### 4.4. Paeonia lactiflora Pall. Pall.

*Paeonia lactiflora Pall. Pall.*, the rhizome of *Paeonia lactiflora Pall. Pall.* from the Paeoniaceae family. In TCM, *Paeonia lactiflora Pall*. is believed to exert the therapeutic effects of nourishing blood and regulating menstruation, astringing yin to arrest sweating, soothing the liver to alleviate pain, and subduing hyperactive liver yang. It has long been employed in traditional medicine for the management of diverse inflammatory disorders and enhancement of immune function, with notable efficacy in alleviating muscle spasms, menstrual cramps, and certain musculoskeletal disorders involving bones and joints. Beyond its traditional uses, *Paeonia lactiflora Pall*. is being explored for the treatment of cancer and endocrine diseases. Modern pharmacological studies have confirmed that the pharmacological activity of *Paeonia lactiflora Pall*. originates from the abundant active components in its rhizomes, among which paeoniflorin serves as the core bioactive constituent. In addition, multiple other active components in *Paeonia lactiflora Pall*. including albiflorin, hydroxypaeoniflorin, benzoylpaeoniflorin, paeonol and flavonoids—can exert synergistic effects to enhance its overall pharmacological activity. As a monoterpenoid glycoside compound, paeoniflorin exhibits favorable water solubility; structural modification via the introduction of functional groups such as acyl and alkyl moieties can simultaneously improve its lipid solubility and water solubility, thereby enhancing its anti-inflammatory and anti-muscle atrophy activities [163,164].

Some studies have indicated that *Paeonia lactiflora Pall*. can mitigate muscle atrophy caused by colorectal malignancies. Administration of *Paeonia lactiflora Pall*. extract at a daily dose of 50 mg/kg in the cachexia model of CT26 tumor-bearing mice effectively ameliorated muscle loss and increased food intake in the mice. This therapeutic effect was mainly by controlling serum levels of inflammatory cytokines like IL-6 and TNF-α, inhibiting the activation of the NF-κB signaling pathway, and improving the systemic inflammatory response. Additionally, *Paeonia lactiflora Pall*. may help improve appetite, though the underlying mechanisms remain unclear. Paeoniflorin, a key active compound in *Paeonia lactiflora Pall*., follows first-order elimination kinetics in humans: the mean terminal t½ is 1.11 ± 0.03 h, steady-state volume of distribution (Vss) is 0.19 ± 0.03 L/kg, and total clearance is 0.12 ± 0.02 L/h/kg, which is approximately 5% of cardiac plasma flow. Renal clearance accounts for 58.3–63.6% of total clearance (CLtot), and the product of renal clearance (CLR) and the free fraction (fu = 82.3%) approximates the human glomerular filtration rate, confirming glomerular filtration as the predominant renal excretion mechanism. After body weight normalization, no gender differences in pharmacokinetics were observed, and repeated dosing for seven days showed no accumulation, with a relative accumulation (Rac) of approximately 0.85 after 7 days [139,165,166].

Although *Paeonia lactiflora Pall*. has exhibited distinct advantages in combating cancer-associated muscle atrophy in animal experiments and preliminary clinical studies, multiple translational bottlenecks remain to be addressed. The animal models adopted in basic research mostly involve muscle atrophy induced by a single tumor type, which diverges from the multifactorial pathological conditions in humans and may therefore undermine the reliability of therapeutic efficacy extrapolation. Additionally, existing clinical studies are constrained by small sample sizes, with a paucity of supportive data from large-scale, multicenter, long-term follow-up phase III clinical trials to substantiate its therapeutic effectiveness.

### 4.5. Panax ginseng C. A. Mey

Ginseng, as defined pharmacognostically, consists of the dried root and rhizome of *Panax ginseng C. A. Mey.* and is widely used in Asia for its tonic effects, particularly for combating fatigue, anorexia, and weight loss from various causes. The primary active components of ginseng are ginsenosides, known for their anti-inflammatory and antioxidant properties. The primary bioactive components of Ginseng are ginsenosides, which exhibit well-documented anti-inflammatory and antioxidant activities. To date, a variety of ginsenosides have been identified, including Rb1 and Rd. In addition to ginsenosides, ginseng polysaccharides and ginseng polypeptides also serve as potent bioactive components of *Panax ginseng*. However, research on the interactions among these multiple components remains scarce, warranting further in-depth investigations [167].

Fatigue, in addition to muscle loss, is a prominent symptom of CC, and studies on ginseng for cancer-related fatigue have shown significant improvement in fatigue symptoms. In terms of ameliorating muscle wasting, Ginseng also exerts a certain effect. In a study conducted on cisplatin-induced cachectic rat models, Ginseng extract was administered at doses of 0, 25 and 50 mg/kg per day. Regarding muscle wasting, ginseng has demonstrated some effectiveness, particularly in elevating the levels of growth differentiation factor (GDF-15) and troponin, which aid in improving muscle mass. However, these mechanisms are still not fully understood, and further in-depth studies are needed to explore the therapeutic potential of ginseng in treating CC. *Panax quinquefolius*, a close relative of *Panax ginseng*, shares similar chemical properties and therapeutic effects. In addition to ginsenosides, *Panax quinquefolius* also contains unique polysaccharides considered to be active ingredients. However, research on the use of *Panax quinquefolius* for CC is currently limited, and this gap remains to be addressed. Pharmacokinetic studies of ginsenosides reveal a Tmax (time to peak concentration) ranging from 2 to 15 h, with dose-proportional Cmax and high inter-individual variability (CV 30–100%). The terminal half-life (t½) ranges from 1 to 12 h. Repeated dosing prolongs the mean residence time (MRT) and results in modest accumulation due to gradual deglycosylation by gut microbiota. Protopanaxadiol ginsenosides, such as Rb1 and Rd, are converted by intestinal microflora into Compound K (CK), with CK’s AUC (area under the curve) strongly correlating with Rd’s AUC (r > 0.8), making CK a useful biomarker for the active metabolite. Various disease states and different processing methods can increase membrane permeability and reduce biliary efflux, leading to a 1.5- to 3-fold increase in AUC. Non-linear absorption is observed for Rd, with a 1.3-fold increase in AUC when the dose escalates from 200 to 2000 mg/kg. Tissue concentrations are highest in the liver, kidneys, and spleen, while plasma protein binding exceeds 80%. Renal excretion accounts for 5–15% of the parent compound, with the remainder eliminated via bile and feces [140,168,169].

Studies on the clinical application of ginseng extract preparations in non-cachectic patients have demonstrated the absence of significant adverse effects, with only a small subset of patients potentially experiencing mild gastrointestinal discomfort. In the future, the development of diverse ginseng extract preparations represents a highly promising strategy for the treatment of cancer cachexia [170,171].

### 4.6. Astragalus membranaceus (Fisch.) Bunge

*Astragalus membranaceus* (*Fisch.*) *Bunge*, a plant in the legume family, yields Astragalus rhizomes, which are frequently used in traditional medicine to address conditions associated with weakness. Astragalus polysaccharide (APS), the primary active constituent, has demonstrated broad pharmacological effects, including anti-inflammatory, oxidative stress-reducing, and anti-tumor properties [172]. Besides astragalus polysaccharides, the roots and rhizomes of Astragalus also contain a variety of bioactive components, including astragaloside IV, flavonoids, multiple amino acids and trace elements; these components exert synergistic effects to enhance the overall pharmacological activity of the herb [173]. APS is a water-soluble heteropolysaccharide. The intensity of its pharmacological activity is correlated with the molecular weight, sugar chain structure and monosaccharide composition of APS; specifically, APS exhibits the strongest anti-inflammatory and immunomodulatory activities when its molecular weight ranges from 20 to 50 kDa [174].

In one study, where a combination of Astragalus and *Angelica sinensis* was administered at respective daily doses of 1, 2.5, and 5 mg/kg, the formulation was more effective in improving CC caused by lung cancer, as it inhibited serum levels of IL-6, IL-4, IL-1, and TNF-α. Additionally, this combination reduced phosphorylation levels of MAPK and NF-κB signaling pathways, helping control CC symptoms. In chronic renal failure rats, astragalus polysaccharide inhibited UPS activation and reduced Atrogin-1 expression. Current research confirms that Astragalus can reduce levels of inflammatory cytokines like TNF-α, IL-8, IL-1β, and IL-32, alleviating inflammation. In one study, astragalus polysaccharide was shown to reduce phosphorylation in the PAK-p38MAPK signaling pathway in a rat model of cardiac CC, preventing fat browning and reducing weight loss. It also lowered IL-6 levels, mitigating inflammatory responses. However, detailed human metabolic data on its effects remain scarce [141,175,176].

### 4.7. Atractylodes macrocephala Koidz

*Atractylodes macrocephala Koidz.*, from the rhizome of *Atractylodes macrocephala Koidz.*, a plant in the Asteraceae family, is commonly used in TCM as a tonic for the spleen and stomach, particularly to treat gastrointestinal dysfunction. Atractylenolide I, a primary active ingredient in *Atractylodes macrocephala Koidz*., is known for its anti-inflammatory, anti-tumor, and metabolism-enhancing properties. In addition to Atractylenolide I, the rhizomes of *Atractylodes macrocephala Koidz.* also contain other bioactive constituents including Atractylenolide II, Atractylenolide III, volatile oil components such as atractylon, atractylol and hinesol, as well as polysaccharides and amino acids. These components exert synergistic effects to enhance the herb’s overall pharmacological activity; for instance, Atractylenolide II and Atractylenolide I can synergistically strengthen anti-inflammatory activity and inhibit lipolysis, while atractylon can assist Atractylenolide I in regulating gastrointestinal function and ameliorating intestinal flora balance [177,178]. Studies have demonstrated that Atractylenolide I is a sesquiterpene lactone compound, and its lactone ring structure serves as the key active site mediating its anti-inflammatory and anti-cachectic properties.

In a CT26 mouse model of CC, it was found that when Atractylenolide I was administered at a daily dose of 25 mg/kg, it reduced weight loss, decreased the phosphorylation of hormone-sensitive lipase (HSL), and prevented excessive lipolysis. Additionally, it inhibited UPS activation, lowered MuRF-1 expression, and elevated MyoD levels to prevent muscle atrophy. Recent research has also highlighted EVs as key drivers of CC, with tumor-derived vesicles delivering materials that exacerbate CC and cancer metastasis. One study demonstrated that Atractylenolide I ameliorates malignant stroma by reducing IL-6 and tumor-derived EV production, mainly by inhibiting the STAT3-PKM2-SNA23 signaling pathway and lowering IL-6 levels. Pharmacokinetic studies in rats indicate that atractylenolides are detected in plasma within 5 min of oral administration, with elimination occurring over approximately 12 h. Tmax is around 0.5 h for Atractylenolide I and II, with Atractylenolide II slightly delayed to 1 h. AUC0–t increases linearly with dose. However, no clinical data are available to confirm these findings or establish efficacy in humans [142,179,180].

Although *Atractylodes macrocephala Koidz*. and Atractylenolide I have demonstrated evident potential in the management of cancer cachexia, current research remains confined to the levels of animal and cellular experiments, and further clinical studies are therefore required to confirm their safety profiles.

### 4.8. Polygonum cuspidatum Sieb. et Zucc.

Polygoni Cuspidati, the root of *Polygonum cuspidatum Sieb. et Zucc.*, contains resveratrol and rhodopsin, both of which have anti-inflammatory and antioxidant properties. Studies have demonstrated that Polygoni Cuspidati, exerts a potent ameliorative effect against CC. Specifically, 20 g of Polygoni Cuspidati extract was incorporated into the daily diet of cachectic model mice for a consecutive 46-day intervention period, whereby the treated mice exhibited marked body weight recovery, a significant elevation in muscle mass, and robust suppression of the adipose browning process. One mechanism through which Polygoni Cuspidati exerts its effects is by upregulating the expression of PTHrP via the TCF4/TIST1 complex, thereby maintaining skeletal muscle homeostasis. Additionally, it regulates two white fat metabolism-related genes—Peroxisome Proliferator-Activated Receptor Gamma Coactivator 1 α (PGC1α) and Acyl-Coenzyme A Oxidase 1 (ACOX1)—to prevent white fat browning and reduce energy consumption. In cancer patients administered a single 5 g oral dose of resveratrol from Polygoni Cuspidati, the parent compound reached a mean Cmax of 1.94 µg/mL, but its absolute bioavailability was less than 1%. The circulating drug consisted primarily of sulfate and glucuronide conjugates, while intratumoral concentrations were higher than those in plasma. Emodin, another compound from Polygoni Cuspidati, has been shown to suppress NF-κB, thereby reducing the transcription of inflammatory cytokines and exhibiting potent anti-inflammatory effects. Additionally, Emodin inhibits T-cell apoptosis and enhances anti-tumor activity. However, Emodin exhibits extremely low oral bioavailability due to extensive Phase-II metabolism, particularly glucuronidation, which rapidly converts the parent compound into undetectable metabolites. Trace amounts of Emodin reach the liver and brain. Inhibiting UDP-glucuronosyl-transferases (e.g., UGT1A8) or co-administering absorption enhancers like piperine significantly increases plasma exposure (Cmax and AUC), but the safety of these metabolic bypass strategies remains uncertain. As a result, conventional oral delivery of Emodin is not optimal, and further research is needed to assess the risk-benefit balance of metabolic inhibition and to develop safer formulation approaches [143,181,182].

### 4.9. Other Herbs

In addition to the repeatedly validated herbs for treating CC, several previously investigated botanicals have also shown promise in CC therapy. Arctii Fructus, the dried seed of *Arctium lappa* L., has been reported to improve CC in both mild and severe CC models. Arctii Fructus extract has exhibited definitive therapeutic efficacy in the CT26 cachexia model. When administered at a daily dose of 100 mg, it significantly attenuated the extent of body weight loss and mitigated muscle and fat wasting in mice, yet exerted no notable ameliorative effect on the animals’ food intake. [144]. *Magnolia officinalis Rehd. et Wils*. has been shown to improve CC, with its active compound, Magnoliae Officinalis Cortexol, reducing levels of inflammatory factors and alleviating the inflammatory response, thereby improving CC. In the cisplatin-induced murine cachexia model, *Magnolia officinalis Rehd. et Wils.* extract, administered at doses of 50, 100, and 200 mg respectively, effectively attenuated muscle loss in mice [145]. *Angelica sinensis (Oliv.) Diels*, when used in combination with *Astragalus membranaceus*, has been reported to improve CC through mechanisms similar to those of *Astragalus membranaceus*, mainly by reducing the levels of inflammatory factors in the body [141,175]. Although these herbs have demonstrated some therapeutic benefits, their mechanisms require further investigation.

### 4.10. Future Perspectives and Cross-Herb Analysis

Although the previous sections have systematically cataloged anti-CC botanicals, a direct comparison is still needed to optimize their clinical applicability. NF-κB blockade emerges as the central theme, with Coix, Scutellaria, Paeonia, and Astragalus all inhibiting p65 activation, suppressing IL-6/TNF-α, and mitigating UPS-driven muscle loss. However, each herb offers a unique contribution: Astragalus dampens all three MAPK pathways and lowers IL-1/IL-4, providing the most comprehensive metabolic reset; Paeonia also inhibits NLRP3 inflammasome assembly, offering multi-target regulation of inflammation; Scutellaria excels at preventing p65 nuclear translocation and delivers the most extensive reduction in cytokines; Coix not only blocks NF-κB but also restrains AMPK-HSL signaling, limiting excessive lipolysis. Muscle-directed strategies complement these effects: Scutellaria, Paeonia, and Atractylodes down-regulate Atrogin-1/MuRF-1; Atractylodes additionally up-regulates MyoD and inhibits STAT3, coupling anti-catabolic and pro-anabolic actions, while Ginseng elevates GDF-15 and troponin to promote myogenesis. Several herbs also exhibit immunomodulatory effects: Magnolia polarizes macrophages toward an anti-inflammatory phenotype, and *Polygonum cuspidatum* increases PTHrP to preserve skeletal muscle mass and mitigate adipose browning. However, clear limitations remain: botanicals rarely target canonical oncogenes like RAS or TP53, meaning their direct tumor-static effects are modest. Additionally, most data are derived from murine xenografts with limited human pharmacokinetic information, and large-scale randomized controlled trials are lacking—representing a principal barrier to clinical adoption. In conclusion, most herbs target CC through a combination of anti-inflammatory and metabolic modulation, exemplifying inherent polypharmacology. The next step is to rationally combine purified active compounds—such as baicalin (NF-κB inhibitor) and atractylenolide (pro-anabolic)—into a single nano-carrier to correct immune-metabolic imbalances while improving bioavailability. However, these combinations must be validated in adequately powered randomized controlled trials to establish safety and efficacy before they can be integrated into mainstream CC care.

## 5. Comparison Between Conventional Treatment and Natural Herbal for Cancer Cachexia

Standard therapeutic modalities for cachexia currently rely primarily on multimodal, multidisciplinary integrated interventions. The pathogenesis of cachexia is highly complex, involving multi-organ crosstalk and overlapping pathological mechanisms. Given this complexity, monotherapeutic approaches are often inadequate to reverse its progressive pathological course, thus mandating the combined implementation of nutritional support, pharmacotherapy, and exercise rehabilitation. Agents commonly prescribed for the clinical management of cachexia include omega-3 fish oil, celecoxib, megestrol acetate, medroxyprogesterone acetate, olanzapine, testosterone, and cannabinoids. The primary pharmacological actions of these agents involve stimulating the central appetite-regulatory circuitry to augment food intake, as well as downregulating the levels of pro-inflammatory cytokines including IL-6 and TNF-α to mitigate chronic inflammation. Additionally, many oncologists routinely recommend progressive resistance training protocols for patients to mitigate the risk of cachexia onset [32,183].

Despite the holistic nature of standard therapies, they are plagued by inherent limitations. For instance, antidepressants such as olanzapine have not been subjected to large-scale randomized controlled trials to validate their efficacy and safety profiles, whereas long-term administration of agents like megestrol acetate is associated with a range of adverse events. Although exercise rehabilitation confers therapeutic benefits, it is poorly tolerated in patients with restricted physical mobility. Furthermore, the single-target specificity of synthetic drugs precludes simultaneous modulation of multiple pathogenic nodes; targeting an individual molecular target fails to disrupt the vicious cycle of inflammation, metabolic dysregulation, and muscle atrophy that underpins cachexia progression. In contrast, the multi-target pharmacology of phytomedicines has been well substantiated, as numerous herbal formulations exert concurrent effects on suppressing catabolic pathways and enhancing anabolic processes. A paradigmatic example is astragalus polysaccharide, a bioactive phytoconstituent isolated from *Astragalus membranaceus*: it modulates gut microbiota to reduce LPS burden, abrogates LPS- TLR4binding, inhibits the NF-κB-mediated inflammatory signaling cascade, and concurrently activates the mTOR anabolic pathway to promote skeletal muscle protein synthesis, thereby achieving multi-dimensional modulation of the core pathological processes in cachexia [184]. This systemic regulatory advantage directly addresses the single-target limitation of conventional treatments. Notably, the integration of standard therapies with phytomedicines can further optimize treatment outcomes. A dedicated Meta-analysis focusing on phytotherapeutic interventions for cachexia has corroborated the scientific rationale underlying this combinatorial strategy. Studies have demonstrated that, compared with the conventional monotherapy cohort, patients receiving combined conventional-phytotherapeutic treatment exhibited significant improvements in appetite, quality of life, and weight maintenance, coupled with a lower incidence of treatment-related adverse events [24,26]. This combinatorial therapeutic paradigm has emerged as a pivotal strategy to overcome the bottlenecks in cachexia management, laying a robust foundation for subsequent clinical translational research. To visually delineate the similarities and disparities between these two therapeutic strategies, we present a comparative summary in Table 2.

## 6. Discussion

This review systematically analyzes and summarizes the mechanisms underlying CC and the natural herbs that can be used in its treatment. CC is a metabolic syndrome associated with malignant tumors and involves multiple mechanisms related to skeletal muscle atrophy and lipolysis [15,32]. Despite gaining increasing attention, the pathogenesis and treatment of CC remain unsatisfactory. Several key signaling pathways, including NF-κB, AMPK, and STAT3, require focused investigation in CC [123,124,125], as they could aid in the identification of biomarkers for adjuvant diagnosis. Moreover, CC involves numerous metabolic pathways across multiple organs and tissues, and further exploration of the connections between these mechanisms is essential for future treatment development. In current clinical practice, a clear dichotomy exists between phytochemicals and synthetic therapeutics. While synthetic drugs may provide rapid symptomatic relief, their adverse effects often preclude prolonged use. Therefore, an optimal approach to CC treatment should aim to restore immune-metabolic homeostasis and interrupt the inter-organ cycles that perpetuate wasting, rather than merely alleviating isolated symptoms such as muscle atrophy or anorexia. Due to their multi-component and multi-target properties, botanical agents offer a systems-oriented strategy that shifts management from symptom relief to causal modulation. Emerging data from early-phase human studies indicate that standardized herbal extracts can reduce chronic systemic inflammation (evidenced by decreases in IL-6 and TNF-α), correct energy dysmetabolism, and simultaneously suppress proteolysis while promoting myofibrillar protein synthesis. This integrated pharmacological profile is unattainable with single-target drugs. However, herbal medicines should not be viewed as simple “green” alternatives to synthetic compounds. Instead, they represent a complementary therapeutic approach whose primary value lies in re-calibrating the immune-metabolic set point. Rigorous validation through large-scale, multicenter, randomized controlled trials is crucial to generate high-level evidence regarding both safety and efficacy, ultimately facilitating the integration of botanicals into evidence-based, precision CC care. Herbal medicines offer several advantages in treating CC, and herbs and herbal tonics are widely used in some Asian countries. However, in-depth mechanistic studies on these remedies remain scarce [133]. This review aims to describe the mechanisms of CC and the proven molecular mechanisms and safety profiles of natural herbal remedies for CC, as well as minimize the adverse effects associated with their use. Some herbs, often used as both food and medicine, can be incorporated into therapeutic diets, which represents the safest approach to reducing the burden on patients [185]. Herbs not currently recognized as medicinal foods present an opportunity for future development, with the potential to isolate single active ingredients to form new drugs for the treatment of CC. Research has shown that these herbs play a critical role in improving the quality of life for CC patients, but their use remains limited, primarily due to a lack of knowledge and reporting on their benefits. Therefore, a multidimensional research program should be initiated to promote the integration of herbs in clinical practice [186,187]. Beyond clinical translation challenges, herbal medicines face inherent chemical complexity. Multi-constituent profiles lead to batch-to-batch variability, potential herb-drug interactions, and incomplete toxicological characterization. These challenges must be addressed to ensure the widespread adoption of herbal remedies.

Current evidence suggests that herbal therapeutics should be positioned within a multi-target immune-metabolic regulatory paradigm. Botanicals simultaneously modulate several key nodes in the immune-metabolic axis, thereby alleviating the effects of CC. Polysaccharides, flavonoids, and saponins reduce pro-inflammatory cytokines, antagonize key signaling pathways such as NF-κB, AMPK, and STAT3, reprogram macrophage polarization, and normalize the function of T-cells and MDSCs, thus restoring immune homeostasis. On the metabolic front, these compounds enhance insulin receptor sensitivity, mitigate insulin resistance, suppress inflammation-driven UPS activation, and downregulate Atrogin-1 and MuRF-1. Additionally, microbiota-derived metabolites, such as short-chain fatty acids, mediate gut–immune axis modulation, further improving systemic metabolism. This integrated approach not only supports the use of herbs in treating CC but also provides a theoretical framework for the design of next-generation, multi-target CC therapeutics [186].

Despite the theoretical promise of the multi-target regulatory mechanisms of phytomedicines in CC therapy, current clinical practice remains dominated by chemically synthesized agents, whose real-world efficacy continues to disappoint. One key reason for the non-approval of several synthetic drugs, such as megestrol acetate, for treating CC by the US Food and Drug Administration is the lack of significant improvements in treatment outcomes. Attempts to combine multiple drugs, including herbal and synthetic agents, have also yielded weak therapeutic effects, and patients are forced to bear additional risks associated with these drugs [185]. However, patients often cannot avoid multiple treatment regimens, and some drugs suffer from low bioavailability and notable side effects. It is well recognized that treating CC requires more than addressing superficial issues like muscle wasting and lipolysis—it necessitates a fundamental improvement in the patient’s quality of life. This highlights the importance of careful medication use to avoid adverse effects. For this reason, focusing on single herbs with clearly defined active ingredients is preferable, as opposed to compounded formulations, which tend to be more complex in composition. Moreover, there is a lack of research on compounded remedies, particularly for CC, and the few studies conducted have not demonstrated better results. This suggests significant limitations in the clinical application of compounded remedies, making the focus on single herbs with well-established compositions both sensible and necessary. Unfortunately, the widespread recognition of herbal remedies is largely confined to East Asia, making it challenging to promote them as first-line or adjunctive treatments for CC globally. It is hoped that this review of herbs will provide patients with viable alternatives to conventional first-line regimens.

## 7. Conclusions

Exploring molecular mechanisms and clinical therapeutic perspectives offers new avenues for drug development for CC. In the future, beyond further research to demonstrate that herbs have low side effects and can achieve the desired therapeutic effects, rather than merely relying on chance, efforts should focus on discovering more effective herbs or isolating active ingredients for clinical treatment to alleviate the suffering of CC patients. This approach will also deepen our understanding of CC. Herbal medicines should not be considered mere “green” substitutes for synthetic drugs. Instead, they should be viewed as a vital complementary therapeutic module to standard-of-care treatments for CC, with unique value in rebalancing immune–metabolic homeostasis. Future research must prioritize high-quality, adequately powered randomized controlled trials to generate solid evidence of both efficacy and safety, supported by real-time pharmacovigilance systems to monitor both beneficial and adverse outcomes. Currently, the body of evidence is largely based on preclinical studies, and species-specific pharmacokinetic differences may limit direct clinical translation. The integration of LC-MS-based quantitative metabolomics with clinical outcomes is essential to map the exposure–response relationship, identify active circulating moieties, and optimize dosing regimens. Network pharmacology, molecular docking, and multi-omics platforms should be utilized to dissect critical nodes within the immune–metabolic network targeted by botanicals, enabling the identification of biomarkers that reliably reflect therapeutic response and guide precision prescribing. Finally, clinical programs should incorporate herb–drug combination therapies within a multidisciplinary framework that also includes non-pharmacological interventions, ensuring a comprehensive, multi-modal approach to CC management.

## Figures and Tables

**Table 1:** Natural herbs for the treatment.

Herb	Experimental Model	Experimental Dose	Sample Size	Biomarker Alterations	Body Weight and Muscle Changes	Compliment	References
*Coix lacryma-jobi* L. *var. ma-yuen (Roman.) Stapf*	Mouse Lewis cachexia Model	*Coicis Semen* extract (*Coicis Semen* oil) 2.5 mL/kg/d gavage	n = 7	Reduced MuRF-1 expression; reduced activation of NF-κB signaling pathway	The body weight of mice in the model group was 16 g, while that of mice in the *Coicis Semen* (coix seed) intervention group increased by 2 g. The cross-sectional area of muscle fibers improved from 700 μm^2^ to 800 μm^2^, with an increase of 100 μm^2^ (*p* < 0.05).	Only one dose group was set up, and negative results were not mentioned.	[136]
*Scutellaria baicalensis Georgi*	Mouse CT26 cachexia model	It was divided into two dose groups, at 150 mg/kg/d and 50 mg/kg/d respectively, while the control group was injected with 200 μL PBS. Drug administration was conducted continuously for 15 days.	n = 12	Reduce Atrogin1, MuRF-1, IL-6, TNF-α expression; reduce activation of NF-κB signaling pathway	The three-day food intake of mice in the model group was 20 g; the food intake of the 150 mg/kg group increased by 12 g, and that of the 50 mg/kg group increased by 10 g. The body weight of the model group on Day 16 was 17 g, while the body weight of the two drug-treated groups increased by 3 g (*p* < 0.05). The muscle weight increased from 178 mg to 232 mg and 228 mg (*p* < 0.05).	Negative results were not mentioned.	[137]
*Coptis chinensis Franch.*	Mouse CT26 cachexia model	*Coptis chinensis* and berberine were mixed into the feed, with the set drug doses of 10 mg/g/d, 20 mg/g/d (for *Coptis chinensis*) and 1 mg/g/d, 4 mg/g/d (for berberine) respectively. Continuous observation was conducted for 14 days.	n = 6	Improvement of feeding, reduction of body weight loss, reduction of IL-1, IL-6	The weight of the gastrocnemius muscle improved from 95 g in the model group to 113 g, 144 g, and 133 g respectively (in the drug-treated groups). In terms of body weight, the model group showed a 5 g decrease from the initial 23 g; in contrast, the drug-treated groups only decreased by 2 g, dropping from 23 g to 21 g.	Negative results were not mentioned.	[138]
*Paeonia lactiflora Pall.*	Mouse CT26 cachexia model	*Paeonia lactiflora* extract, 50 mg/kg/d	n = 8	Improve diet, reduce Atrogin1, MuRF-1, IL-6, TNF-α expression; reduce activation of NF-κB signaling pathway	The body weight of the model group was 23 g; compared with the model group, the body weight of the drug-treated group increased by 1 g.	Only one dose group was set up, and negative results were not mentioned.	[139]
*Panax ginseng C. A. Mey.*	Cisplatin-induced cachexia model in rats	Ginseng extract 25 mg and 50 mg/kg/d	n = 17	Improvement of feeding and malaise, reduction of muscle loss	By day 35 of ginseng administration, body weight had increased by 30 g.	Negative results were not mentioned.	[140]
*Astragalus membranaceus (Fisch.) Bunge*	Lewis cachexia model in mice	1 mg, 2.5 mg, 5 mg/kg/d Astragalus and *Angelica sinensis*	n = 10	Inhibit serum levels of IL-6, IL-4, IL-1, and TNF-α, reduce phosphorylation of MAPK, NF-κB signaling pathway	The muscle cross-sectional diameter increased by 5 μm. The dietary intake was increased (1 mg group: 32.2 g/d, 2.5 mg group: 28.1 g/d, 5 mg group: 32.7 g/d, *p* < 0.015).	Negative results were not mentioned.	[141]
*Atractylodes macrocephala Koidz.*	Mouse CT26 cachexia model	25 mg/kg/d Atractylenolide	n = 7	Reduced weight loss, decreased expression levels of IL-6, MuRF-1 and elevated MyoD expression levels	After 18 days of drug administration, the body weight of the model group was 22 g, while that of the drug-treated group was 24 g, which was an increase of 2 g compared with the model group. The muscle cross-sectional area increased from 120 μm^2^ to 180 μm^2^.	Only one dose group was set up, and negative results were not mentioned.	[142]
*Polygonum cuspidatum Sieb. et Zucc.*	Mouse A549 cachexia model	Mice were provided with feed containing 2% emodin ad libitum.	n = 5	Inhibition of PTHLH and PTHrP expression, along with reduction of Atrogin1 and MuRF-1 expression.	The muscle weight in the model group was 725 mg, while in the emodin-treated group it was 800 mg, representing an increase of 75 mg.	Negative results were not mentioned.	[143]
Arctium lappa* L.	Mouse mild and severe CT26 cachexia model	Arctii Fructus 100 mg/d	n = 10	Reduces IL-6 levels and decreases the expression of Atrogin1 and MuRF-1.	The model group had a body weight of 23 g, which was only 12%, 12.61%, and 14.23% lower than that of the treatment groups, respectively.	Negative results were not mentioned.	[144]
*Magnolia officinalis Rehd. et Wils.*	Cisplatin-induced cachexia model in mice	Combination of 50, 100 and 200 mg of *Magnolia officinalis Rehd. et Wils*. extract cisplatin injection	n = 10	Upregulated the levels of macrophage M2 markers (CD206, Arg-1, TGF-β, and CD163) and increased the level of IGF-1.	The hindlimb weight in the control group was 0.8 g, and the treatment groups showed increases of 0.1 g, 0.2 g, and 0.3 g, respectively.	Negative results were not mentioned.	[145]
*Angelica sinensis (Oliv.) Diels*	Lewis cachexia model in mice	1 mg, 2.5 mg, 5 mg/kg/d Astragalus and *Angelica sinensis*	n = 10	Inhibit serum levels of IL-6, IL-4, IL-1, and TNF-α, reduce phosphorylation of MAPK, NF-κB signaling pathway	Muscle cross-sectional diameter increased by 5 μm. Food intake was increased (1 mg group: 32.2 g/d; 2.5 mg group: 28.1 g/d; 5 mg group: 32.7 g/d; *p* < 0.015).	Negative results were not mentioned.	[141]

**Table 2:** Summary Table of Comparison between Conventional Treatment and Natural Herbal for Cancer Cachexia.

Comparison Dimension	Conventional Treatment of Cancer Cachexia	Natural Herbal Medicines
Regulation Mode	Single-point intervention, targeting only one pathological link of cachexia (e.g., central appetite, inflammation, nutritional supplementation), failing to cover the multi-organ interaction network	Systematic regulation, covering a large area at the same time, including intestinal barrier reconstruction, systemic inflammation inhibition, increase in muscle protein synthesis, appetite control, and so on, covering all key nodes of the “gut-immune-metabolism-muscle” axis
Therapeutic Target Spectrum	Single target (e.g., celecoxib only acts on COX-2; anti-IL-6 antibodies only bind to IL-6 receptors), making it difficult to address cross-disorder of multiple pathways	High safety, low incidence of adverse reactions, which are mostly mild and mainly gastrointestinal symptoms, can be relieved by reducing the dose or discontinuing the drug; most of the herbal medicines are of medicine-food homology and can be used for long-term intervention of cachexia patients
Safety	High incidence of adverse reactions, most of which are severe (e.g., thromboembolic risk of megestrol acetate, gastrointestinal injury from celecoxib), limiting long-term use	High safety and low frequency of adverse reactions that are mild (mainly gastrointestinal discomfort), can be relieved by reducing the dose or discontinuing the drug; most herbal medicines are of medicine-food homology, suitable for long-term intervention on cachexia patients
Modulatory Effect on the Core Pathological Cycle	Unable to break the core cycle of “inflammation—metabolic disorder—tissue depletion” (e.g., although celecoxib can inhibit inflammation, it cannot repair the intestinal barrier, making it difficult to prevent recurrent inflammation caused by continuous LPS entry into the bloodstream)	Can block important links of the core cycle through synergistic effects on different targets such as *Astragalus membranaceus* decreases LPS translocation by adjusting the intestinal flora (cut off inflammation source), inhibit NF-κB inflammatory pathway (stop inflammation in the middle), and activate the mTOR anabolic pathway (protect muscle downstream), break the vicious cycle on different levels

## Data Availability

Not applicable.
